# Platelets involved tumor cell EMT during circulation: communications and interventions

**DOI:** 10.1186/s12964-022-00887-3

**Published:** 2022-06-03

**Authors:** Xiaoying Wang, Songyan Zhao, Zhaoxia Wang, Tao Gao

**Affiliations:** 1 Jiangsu Key Laboratory for Molecular and Medical Biotechnology, College of Life Sciences, Nanjing Normal University, Nanjing, 210023 People’s Republic of China; 2 Department of Oncology, The Second Affiliated Hospital of Nanjing Medical University, Nanjing, 210011 People’s Republic of China; 3 Department of Oncology, The Affiliated Changzhou No. 2 People’s Hospital of Nanjing Medical University, Changzhou, 213100 People’s Republic of China

**Keywords:** Platelet, Circulation tumor cell, EMT, Hematogenous metastasis, Signal communications

## Abstract

Distant spreading of metastatic tumor cells is still the leading cause of tumor death. Metastatic spreading is a complex process, in which epithelial-mesenchymal transition (EMT) is the primary and key event to promote it. Presently, extensive reviews have given insights on the occurrence of EMT at the primary tumor site that depends on invasive properties of tumor cells and the tumor-associated microenvironment. However, essential roles of circulation environment involved in tumor cell EMT is not well summarized. As a main constituent of the blood, platelet is increasingly found to work as an important activator to induce EMT. Therefore, this review aims to emphasize the novel role of platelet in EMT through signal communications between platelets and circulation tumor cells, and illustrate potent interventions aiming at their communications. It may give a complementary view of EMT in addition to the tissue microenvironment, help for better understand the hematogenous metastasis, and also illustrate theoretical and practical basis for the targeted inhibition.

**Video abstract**

**Supplementary Information:**

The online version contains supplementary material available at 10.1186/s12964-022-00887-3.

## Background

Uncontrollable proliferation, invasion and metastasis of cells are not only the basic characteristics of malignant tumor [1–3], but also the primary cause of tumor-related mortality. Tumor invasion and metastasis are complex and multi-stage processes, in which tumor cells transit and migrate from the primary tumor site into the blood circulation, survive in it, adhere and migrate across vascular endothelium, and finally proliferate and develop into visible metastatic sites. Different traits that equip tumor cells with the ability to leave and travel are acquired at different stages during this process [4, 5]. Epithelial-mesenchymal transition (EMT) is a primary and highly regulated process that plays key roles in tumor cell invasion. Epithelial tumor cells acquire mesenchymal motility and migration ability through a series of coordinated molecular events and cellular changes [6, 7]. Signal communications between tumor cells and their microenvironment is extremely crucial for EMT [8, 9]. Plenty of studies have shown various signals from tumor microenvironment directly regulate the occurrence of EMT [10–12]. The specific regulatory molecules of EMT in the primary tumor microenvironment have been summarized in detail [10, 13, 14]. In addition, studies have found that blood cells such as neutrophils, macrophages and lymphocytes can be recruited to tumor microenvironment to participate in EMT [15–18]. However, the mechanism of EMT during circulation has not been systematically described.

Platelets are the major promoter of EMT in circulation. As a kind of non-nuclear blood cells produced by megakaryocytes [19, 20], platelets are not only involved in hemostasis and thrombosis, but also interact with tumor cells after they infiltration into the vasculature [21]. The prerequisite for metastasis is that tumor cells can survive in circulation, which is ascribed to the cross-linking between platelets and tumor cells to a great extent [22]. A variety of steps in tumor metastasis cascade, escaping the immune surveillance, EMT, adhesion to vascular endothelium, trans-endothelial migration and neoangiogenesis, are also closely related to platelet-tumor cell interaction [23–25]. Here, we have focused on the decisive role of platelets in EMT during circulation, and reviewed signal communications and EMT-promoting mechanisms by platelet, which not only helps to better understand the hematogenous metastasis, but also provides theoretical and practical basis for the targeted inhibition.

## Platelet activation induced by tumor cells

Platelets are small in size with a diameter about 2–5 μm and tend to be marginalized to the outer edge of blood flow in vessels [26]. The cytoplasm of platelet contains a large number of α granules and dense granules. α granules are rich in membrane-bound and soluble proteins, which are engaged in platelet adhesion, coagulation, angiogenesis and immune cell recruitment. Dense granules contain platelet agonists such as ADP and serotonin, which are involved in platelet activation and aggregation [27].

**Scheme 1:**
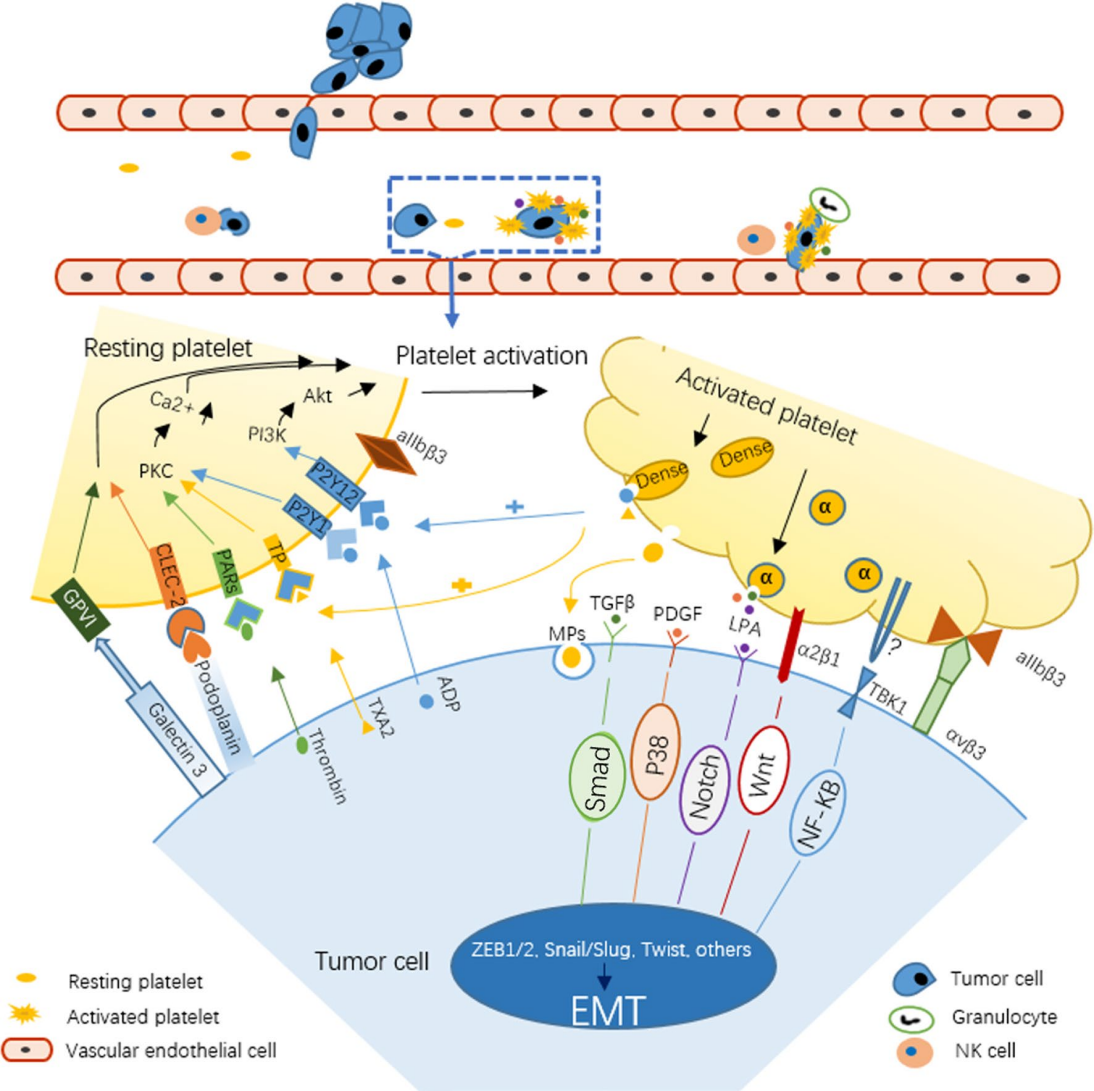
Schematic illustration of the interactions between platelet and tumor cell in the circulation system to promote EMT

After tumor cells invade into blood circulation, they first interact with the marginalized platelets [28]. Tumor cells exploit various mechanisms to induce platelet activation (Scheme 1). They can release soluble platelet agonists such as ADP, thromboxane A2 (TXA2), or thrombin, which bind to platelet receptors P2Y1 and P2Y12, thromboxane-prostanoid (TP), and proteinase-activated receptors (PARs) respectively. This adhesion activates G protein-coupled receptors signaling pathway and eventually leads to conformational change of membrane integrin aIIbβ3 from low-affinity to high-affinity [27]. The high-affinity integrins on activated platelets can form a firm adhesion with tumor cells, just like a "platelet cloak", which is believed to facilitate the survival and metastasis of tumor cells in circulation [29–32]. Tumor cells can also express binding proteins (such as podoplanin, PDPN) which can bind to platelet surface adhesion proteins (such as C-type lectin-like receptor 2, CLEC-2) to promote platelet activation [33]. Upon platelet activation, intracellular granules rapidly fuse with plasma membrane. Membrane-bound proteins from α granules are exposed to the platelet surface, and soluble proteins are released into the extracellular microenvironment [34–36]. Platelet agonists in dense particles, such as ADP and TXA2, will act as positive feedback mediators to further induce platelet activation [35].

## Activated platelets promote tumor cell EMT

The combination of tumor cells and activated platelets forms the initial metastasis niche, and a series of changes of activated platelets mediate the occurrence of EMT [37, 38]. In addition to platelets, there is the deposition of fibrin in the "platelet cloak" [22], thus forming a local microenvironment that maintains a relatively high concentration of EMT signals. Series of biological effects are realized by a variety of secreted factors from activated platelets. Some proteins expressed by platelets have also been reported to involve EMT by promoting the release of these secretory factors. These complex signal communications are discussed together in detail in the following sections.

### Transforming growth factor beta (TGFβ)

In 2011, Labelle et al. found that the interaction between tumor cells and platelets can induce tumor cell EMT and promote metastasis. Tumor cells (colon cancer cell line MC38GFP and breast cancer cell line Ep5) incubated with platelets were injected into mice. Compared with tumor cells without incubation with platelets, the number of lung metastasis foci increased significantly, suggesting that the interaction between tumor cells and platelets in vivo directly promoted tumor metastasis. Meanwhile, morphological changes and increased invasiveness were observed in tumor cells incubated with platelets. Further analysis has showed that the mRNAs of Snail, vimentin, fibrin and plasminogen activator inhibitor-1 were continuously upregulated in platelet-treated cells, while epithelial markers E-cadherin and Claudin1 (Cldn1) were down-regulated. N-cadherin was relocated from the cell–cell junction to the cytoplasm and the secretion of matrix metalloproteinase-9 increased, confirming that platelet induced EMT in tumor cells. Further studies have shown that TGFβ/Smad pathway can be activated by platelet-secreted TGFβ1 and NF-κB pathway can be triggered by direct contact between tumor cells and platelets, and the latter can enhance the TGFβ1 transcriptional response of tumor cells. Ultimately, the two synergistically induce a rapid EMT [39]. When TGFβ1-deficient platelets were incubated with tumor cells, no significant increase in metastasis foci was observed, suggesting that platelet-derived TGFβ1 is essential. Platelets are the main source of TGFβ1 in circulation. It has been reported that its TGFβ1 content is 40–100 times than that of other cells [40]. Therefore, tumor cells wearing a "platelet cloak" can directly perceive platelet-derived TGFβ signal and thus response accordingly. EMT induced by platelet-derived TGFβ has also been confirmed in ovarian cancer cells [41].

Labelle's research opens the door to a new promoting metastasis effect of platelet. However, the mediator of NF-κB pathway activation caused by direct contact between platelets and tumor cells has not been identified [42]. NF-κB activator TANK-binding kinase 1 (TBK1) is a serine/threonine kinase with an ability to induce NF-κB signal and is highly expressed in a variety of tumor cells [43]. When breast cancer cells were co-cultured with platelets, morphologic and molecular change characteristics of EMT were observed and the level of TBK1 activation also increased. Depletion of TBK1 using interfering RNA inhibited platelet-induced EMT formation. The metastasis is also inhibited when TBK1-silenced tumor cells are incubated with platelets, suggesting that TBK1 is a key mediator in regulating platelet-induced EMT [42]. Nevertheless, the platelet ligand binding to TBK1 is unclear and needs to be further confirmed. Direct contact between platelets and MCF-7 through integrin α2β1 has also been observed in breast cancer, which not only activates the Wnt-β-catenin pathway and promotes the expression of EMT-related proteins, but also synergistically facilitates EMT by inducing the TGFβ1 autocrine from tumor cells [44]. These results indicate that in addition to the primary site, tumor cells can respond to platelet-derived EMT signals in the circulation to enhance metastatic potential.

Some factors expressed by platelet have also been shown to mediate EMT by promoting TGFβ secretion. CLEC-2 is selectively and highly expressed in human platelets and megakaryocytes [45]. PDPN is the receptor of CLEC-2, which has been confirmed to be expressed in squamous cell carcinoma, brain tumor, osteosarcoma and melanoma [46]. The expression of PDPN in tumor cell, especially in invasive edge cells, is bound up with poor prognosis in esophageal squamous cell carcinoma patients [47]. The combination of PDPN and CLEC-2 can induce platelet activation and thus promote tumor EMT by platelet-secreted TGFβ, while TGFβ neutralizing antibody can effectively inhibit PDPN-mediated distant metastasis [48]. It has also been reported that the binding of CLEC-2 and PDPN can stimulate the release of sphingosine 1-phosphate (S1P) from platelets [49], which also has the ability to induce EMT [50]. Lack of PDPN significantly reduces cell motility and EMT-related aggressiveness in esophageal squamous cell carcinoma cells, demonstrating that PDPN is crucial for platelets-induced EMT [51].

PAR is a key receptor that regulates platelet activation. There are four types of PAR: PAR1-4, among which PAR1 and PAR4 are expressed on human platelets and can be activated by thrombin [52]. A dose-dependent increase in TGFβ secretion and a decreased expression of miR-200b, an important EMT mediator, was observed in colon cancer cells incubated with activated platelet supernatant induced by PAR1 agonist, indicating that PAR1-activated platelets can induce EMT without direct contact with colon cancer cells [53]. It is noteworthy that a variety of tumor cells also express PAR1. In liver cancer, PAR1 was observed to mediate EMT through Twist1 [54]. In breast cancer, Twist transcriptionally induces the expression of PAR1, which promotes EMT by inhibiting Hippo pathway [55].

P2Y12 is a G protein-coupled receptor on platelets, which participates in ADP-mediated platelet activation and aggregation. P2Y12 deficiency decreases the ability of lung cancer cells to trigger platelet shape change and release TGFβ, therefore leading to diminished platelet-induced EMT. It has also been observed in melanoma cells that both tumor cell-induced platelet aggregation (TCIPA) and platelet-induced EMT are in a P2Y12-dependent manner, and the lack of P2Y12 leads to a significant reduction in lung metastases [56]. The EMT promoting effect of PAR and P2Y12 further confirms that platelet activation is the basis of platelet-involved EMT and tumor metastasis cascade in circulation.

### Platelet-derived growth factor (PDGF)

PDGF is another critical EMT driver that contributes to cancer invasion. The PDGF family consists of four structurally related polypeptide chains that constitute five functional homo-or-heterodimers: PDGF-AA, PDGF-BB, PDGF-AB, PDGF-CC, and PDGF-DD [57]. By binding to cell surface tyrosine kinase receptors (PDGFRα or PDGFRβ), these dimers exert biological effects [58]. It was reported that PDGF-AA and PDGF-CC mainly bind to PDGFRα, and PDGF-BB and PDGF-DD bind to PDGFRβ [57]. PDGF signal has been proved to promote EMT in multiple cancer types [59, 60].

Platelet-derived PDGF can induce EMT of cholangiocarcinoma cells by activating P38 /MAPK signal and up-regulating the expression of MMP2/MMP9 [61]. Exogenous PDGF-D has been identified to up-regulate tumor PDGFRβ expression in tongue squamous cell carcinoma, which promotes EMT through P38/AKT/ERK pathway [62]. Moreover, PDGF-D released by tissue-resident stem cells is responsible for EMT in breast cancer cells and this effect can be neutralized by PDGF antibody [63]. It should be noted that a variety of tumor cells can also express PDGFs and PDGFRs to function in an autocrine way [59, 64]. PDGF-D overexpression of PC3 prostate cancer cells contributes to EMT through activation of mTOR and NF-κB pathway [65] and downregulation of the miR-200 [66]. In endometrial cancer, both in vitro and in vivo experiments have showed that PDGF-D induces EMT through upregulating MMP2/9 [67]. Furthermore, PDGF-D promotes EMT transformation in colorectal cancer via activation of Notch1/Twist1 pathway [68]. In addition to PDGF-D, PDGF-B not only contributes to EMT in gastric cancer cells by activating MAPK/ERK pathway [69], but also promotes the expression of zinc finger E-box binding homeobox 1 (ZEB1) by downregulating the expression of miR-200 in triple negative breast cancer [70].

Interestingly, there is a cross-link between PDGF and TGFβ signal to promote EMT. TGFβ/Smad signal has been identified to promote the growth of malignant glioma cells by inducing PDGF autocrine and paracrine [71], in which ZEB1 is the key regulator for PDGFRα-driven EMT [72]. The interference with PDGF signal reduces TGFβ-induced migration and tumor growth in hepatocellular carcinoma [73]. This conclusion has also been confirmed in breast cancer and colorectal cancer. Breast cancer cells in TGFβ-induced EMT state express an autocrine PDGF/PDGFR loop and continuous autocrine signal is involved in the maintenance of EMT state. Moreover, inhibition of PDGFR signal not only impaired EMT but also led to apoptosis [64]. PDGFRβ was co-expressed with TGFβ and EMT related genes in colorectal cancer cells. Inhibition of TGFβ signal significantly reduced PDGFβ expression level and PDGF-stimulated tumor cell invasion ability, which demonstrates that PDGFR may be a downstream signal of platelet activation and TGFβ signaling [74]. Obviously, EMT driver molecules can activate multiple signaling pathways and the signal crosslinking between different driver molecules co-regulates EMT.

Platelet-derived α particles release abundant PDGFs, and whether tumor cells respond depends on whether they express PDGFR. Breast cancer cells undergoing EMT were found to express elevated levels of PDGFR [75]. Transcription factor Twist1 is known to induce EMT, and Twist1 also induces PDGFRα expression. Meanwhile, PDGFRα is the direct transcription target of Twist1. Both of them are central mediators of invasive foot, which mediates extracellular matrix degradation during metastasis [76]. The expression of PDGFR is closely associated with the invasive phenotype of breast cancer [64]. Derived from both platelets and tumor cells, PDGF binds and activates PDGFR in tumor cells to initiate various biological behaviors. The effect of different secretion patterns on the response of tumor cells needs further verification.

As the receptor for collagen and fibrin, transmembrane protein glycoprotein VI (GPVI) has also been identified to mediate EMT by promoting PDGF secretion. GPVI is only expressed on platelets and megakaryocytes and involved in platelet activation [28]. In colon cancer cells, the binding of GPVI to galectin 3 on the surface of tumor cells can induce platelet activation and therefore secrete PDGF. By combining with PDGFR, PDGF promotes the upregulation of COX2 and the release of prostaglandin E2 from tumor cells. Induction of transcription factors ZEB1 and Twist1 are further detectable, which ultimately contributes to tumor cell transition from an epithelial to a mesenchymal phenotype. This biological effect can be interrupted by COX-2 inhibitor treatment [28, 77], suggesting that GPVI-induced COX-2 expression may be a key mediator of EMT in colon cancer cells.

### Lysophosphatidic acid (LPA)

Platelets are the main source of LPA. Autotaxin (ATX) is a glycosylase that participates in modulating the level of LPA in plasma and has unique phospholipase D activity, which can catalyze a series of lysophospholipid precursors to generate LPA [78, 79]. LPA has six different receptors (LPAR 1-6) that mediate diverse biological functions such as cytoskeletal rearrangement, motility, cytokine secretion and cell differentiation [80]. Activated platelet release ATX and LPA, and LPA levels are significantly elevated based on the further catalytic capacity of ATX. In addition to platelets, tumor and tumor stroma are also the major sources of ATX [80]. It has been reported that binding of LPA to tumor cells LPAR1 promotes tumor invasion, and the combination of LPA with platelets LPAR5 regulates platelet aggregation [81].

Tumor cells in EMT state are accompanied by up-regulation of LPAR mRNA levels and increased responsiveness to LPA [82]. Some tumor cells produce LPA to promote invasion and platelet aggregation. LPAR has been observed in prostate cancer cells to form heterodimers with tumor antigen CD97 to amplify LPA-mediated signal transduction, while CD97 is an adhesive G-protein-coupled receptor that accounts for platelet activation [83]. In gastric cancer cells, LPA was observed to induced EMT by binding to LPAR2 and activating Notch signaling pathway [84]. Receptor for advanced glycation end products (RAGE), a multi-ligand transmembrane receptor of the immunoglobulin superfamily, has been observed to bind LPA and modulate LPA-mediated EMT in lung and breast cancer [85]. High platelet activation-inducing characteristics are closely related to increased expression of LPAR1 in osteosarcoma cells, which binds LPA and invites enhanced invasion ability and lung metastasis. Conversely, knockout of LPAR1 gene or oral LPAR1 antagonist can inhibit lung metastasis, illustrating the significance of LPA-LPAR1 axis in tumor invasion and metastasis [86].

### Platelet-derived microparticles (PMPs)

In addition to the factors mentioned above, activated platelets also release microparticles (MPs). MPs are membrane vesicles secreted by cells, and at least 45% of plasma MPs come from platelets [87]. PMPs not only express p-selectin, integrin αIIbβ3 and other activated platelet-related proteins, but also contain a large number of growth factors, cytokines and miRNAs [88].

Plantureux found that the interaction of platelets and colorectal cancer cells induced the generation of three different MPs: platelet-derived MPs (76%), tumor cell-derived MPs (16%), and mixed MPs (8%) with both platelet and tumor-cell features, which were uniformly named iMPs. Studies have found the existence of iMPs in colorectal cancer tissues, which recruit macrophages through chemokines such as CCL2 and CXCL12, and activate macrophages through IFN γ and IL4 to kill tumor cells, thus inhibit the growth of primary tumors. While in blood circulation, iMPs can activate endothelial cells and platelets, promote EMT of tumor cells and enhance their adhesion abilities. The occurrence of EMT may bound up with the mRNA transferred from platelets, since some mRNAs that are absent in cancer cells alone (mRNA PTGS2 for example, coding for COX2) have been found to be overexpressed in cancer cells primed with platelets [89]. The invasion ability of breast cancer cells incubated with PMPs was significantly enhanced [90]. It has also been observed that PMPs promote tumor metastasis and angiogenesis in lung cancer cell lines by stimulating cell proliferation, increasing expression of angiogenic factors and endothelial growth factors, and enhancing adhesion to endothelial cells [91]. However, above studies do not further clarify the specific mediators that play roles in PMPs. Different stimulation may produce PMPs with different components [88], which may consequently have different effects [5]. It is found in lung cancer cells that miR-233 delivered by PMPs promotes tumor invasion by targeting tumor suppressor EPB41L3 [92]. PMPs can also infiltrate both human and mouse solid tumors and transfer miR-24 to tumor cells, causing mitochondrial dysfunction, growth inhibition and ultimate apoptosis [93]. These results indicate that the specific effects of PMPs on tumor cells are likely to be associated with the local microenvironment, the stimulation type of activated platelets and the composition of MPs.

Enhanced platelet activation has also been identified to promote EMT. Histidine-rich glycoprotein (HRG) is a single-chain heparin-binding plasma protein that specifically binds activated platelets to inhibit their activity. HRG-deficient mice have showed increased platelet count and activation. HRG deficiency promotes tumor cell EMT and angiogenesis. Therefore, HRG can function as a tumor suppressor to inhibit EMT by regulating platelet activity [94]. Interestingly, consensus molecular subtype 4 (CMS4), a molecular subtype closely associated with platelet activation in colorectal cancer, is characterized by EMT, matrix remodeling, angiogenesis, inflammation, stromal infiltration and poor prognosis. It does not mean that EMT is widespread in CMS4 tumor, but rather that tumor cells can recruit platelets through local inflammation and EMT can easily be induced by platelets [95].

In summary, platelets are closely related to EMT in the circulation. The "platelet cloak" and secreted molecules form a microenvironment that are conducive to EMT and tumor invasion. Enhanced mobility, the benefit from EMT, provides tumor cells with a convenient to migrate through the vascular endothelial cells and thereby facilitates the metastasis cascade [22]. However, platelets do not only promote EMT in the circulation. An immune-histochemical analysis has been conducted on 40 pancreatic cancer tissue samples, which has showed that at the invasive front of tumor, the expression of platelet marker CD42b was positively correlated with Snail expression (*P *= 0.02) and negatively correlated with E-cadherin expression (*P* = 0.008), indicating that extravasation platelets aggregation in the primary tumor is related to EMT formation [96]. Abnormal tumor vessels provide the structural basis for platelet extravasation, which may explain the presence of platelets in the primary tumor.

## Targeted inhibition of platelet-tumor interaction

**Scheme 2:**
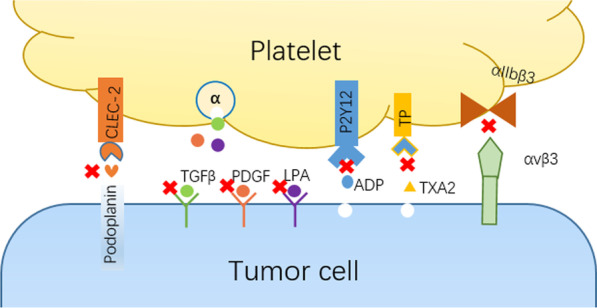
Schematic illustration of the targeted inhibition of the platelet-tumor interaction

Activated platelets are not only involved in tumor EMT, invasion, and metastasis, but also invite venous thromboembolism (VTE), which is the second leading cause of death in tumor patients [97]. Based on the significant role of platelets, targeted blocking of platelet-tumor cell interaction has become a research hotspot, which includes inhibition of platelet activation, platelet receptor-ligand binding and signaling pathways that mediate EMT (Scheme 2).

### TGFβ inhibitor

TGFβ is a multifunctional cytokine that not only participates in tumor cell EMT, but also engages in proliferation, angiogenesis and immunosuppression. High levels of TGFβ are closely tied up with early recurrence, treatment resistance and poor prognosis in tumor patients [98, 99]. The antitumor activity of TGFβ inhibitors has been elegantly reviewed [100]. In general, despite the support of preclinical studies and theoretical basis, it is still challenging to translate it into clinical practice. Combination therapy based on TGFβ inhibition and screening the subgroups of patients who can benefit are the main research directions.

### PDGF-PDGFR pathway inhibitor

Aptamers and monoclonal antibody targeting PDGFR have been developed to block the PDGF-PDGFR pathway. Aptamers have shown antitumor activity in cells and mouse models, but clinical studies have not yet been reported [101, 102]. Olaratumab, a recombinant human PDGFRα antibody, has been shown to extend median overall survival by 11.8 months in combination with doxorubicin for advanced soft tissue sarcoma in a phase II study [103]. However, this clinical benefit was not confirmed by the further phase III study [104].

### LPA-LPAR pathway inhibitor

The anti-metastasis effects of LPAR antagonists have been observed in several preclinical studies. Ki16425 can inhibit the proliferation and migration of lung cancer cells by targeting LPAR1 [105]. Debio 0719s is an oral LPAR1 antagonist that suppresses the metastasis and proliferation of breast cancer cells in mice, but has no effect on the growth and angiogenesis of primary tumors [106]. Specific antagonist of LPAR5 can reduce the proliferation and migration of thyroid cancer cells [107].

### CLEC-2-PDPN pathway inhibitor

The combination of CLEC-2 and PDPN can induce platelet aggregation and strengthen tumor metastasis. PDPN antibody was identified to inhibit platelet aggregation and PDPN-induced lung metastasis in mouse model of lung squamous cell carcinoma [108]. Cobalt porphyrin is a small molecule inhibitor that can bind to CLEC-2 with high affinity, thereby competitively blocking the interaction between CLEC-2 and PDPN. It has been observed in mice that cobalt porphyrin can significantly reduce pulmonary metastasis and thrombosis without increasing the bleeding tendency [109], suggesting that CLEC-2-PDPN blockade may be a promising anti-tumor strategy.

### ADP binding receptor P2Y12 inhibitor

Both activated platelets and tumor cells release ADP, which promotes tumor-induced platelet aggregation. ADP binding receptor P2Y12 inhibitors, such as clopidogrel and ticagrelor, hinder platelet activation and reduce TCIPA by preventing ADP from binding platelets [110, 111]. Preclinical researches have shown that P2Y12 inhibitors can reduce metastasis in melanoma, ovarian, breast, lung, and pancreatic cancers [56, 112–115]. In pancreatic cancer cells, P2Y12 has been found to be specifically expressed and confer a proliferative advantage on tumor cells, and clopidogrel treatment can inhibit the formation of cancer-associated-thrombosis and tumor metastasis [110], suggesting that the blocking of P2Y12 has a dual antitumor effect on pancreatic cancer cells.

### Adhesion receptor integrin αIIbβ3 inhibitor

As the most abundant receptor on platelets, αIIbβ3 can bind to αvβ3 expressed on the surface of tumor cells [116]. Studies in vitro have shown that heparin can block the adhesion receptors integrin αIIbβ3 [117]. Heparin has been observed to weaken the interaction between tumor cells and platelets and reduce the release of platelet-derived mediators, which accordingly lead to decreased expression of EMT marker proteins and transcription factors, therefore ultimately resulting in a marked decrease in cell migration [118].

### TXA2 inhibitor

TXA2 is one of the initiators of platelet activation and its inhibitors have been proved to be effective. As a typical representative, aspirin inhibits the generation of TXA2 by inhibiting cyclooxygenase-1 (COX1), thus inhibiting platelet activation and aggregation. Aspirin can also suppress cyclooxygenase-2 (COX2), which is overexpressed in many tumor cells, including colorectal cancer, breast cancer, gastric cancer and pancreatic cancer. The increased expression of prostaglandin E2 in tumor cells through COX2 is beneficial to tumor proliferation, and aspirin has anti-tumor effect by reducing the level of prostaglandin E2 [37]. Different doses of aspirin have different pharmacological properties. Low-dose aspirin preferentially inhibits COX1 in platelets, while high-dose aspirin can simultaneously inhibit COX1 and COX2 [119]. Aspirin (75–300 mg/d) reduced the risk of distant metastasis and death in patients with adenocarcinoma, and increased dose did not show additional benefit [120], suggesting that the main anti-metastasis target of aspirin is COX1. Inhibition of the COX1/TXA2 pathway in platelets has been proven to reduce platelet aggregation, endothelial activation, tumor cell-endothelial adhesion, and pre-metastasis niche formation. In tumor-bearing mice with platelet depletion, only infusion of platelets with COX1 activity can restore metastasis, indicating the importance of COX1/TXA2 for tumor cells [119]. Aspirin has also been shown to inhibit tumor metastasis and angiogenesis in mouse models by inhibiting heparanase activity [121].

Multiple studies have shown that aspirin can reduce the risk of colorectal cancer, esophageal cancer, gastric cancer, hepatobiliary tumor and pancreatic cancer. Meanwhile, the duration of aspirin use is inversely proportional to the risk of cancer, although such benefit was not observed in head and neck tumors [122–125], demonstrating the heterogeneity of different tumors. It has been observed in colorectal cancer that a dose of 75–100 mg/d can reduce the risk of tumor occurrence by 10%, 325 mg/d can reduce 35%, and 500 mg/d can reduce 50% (although the data for 500 mg/d are limited), suggesting that this preventive effect is dose-dependent [126]. Although high-dose aspirin can inhibit COX2, and COX2 signaling pathway is involved in the occurrence and angiogenesis of colorectal cancer [126], sustained inhibition of COX2 expression in tumor cells requires a higher dose (650 mg, 3–4 times/day) [127]. The most common adverse effects of aspirin are gastrointestinal reactions and bleeding. Therefore, the clinical benefits and risks should be evaluated comprehensively. An increased risk of advanced solid tumors was observed in a healthy population aged ≥ 70 years taking low-dose aspirin daily with an average follow-up of 4.7 years, which may be contributed to the delayed benefit and suppression of anti-tumor immunity of aspirin [128], or to the increased tumor risk in the elderly. This study suggested that the benefit population of aspirin still needs to be further confirmed.

Currently, there is no clinical benefit evidence of aspirin in patients with advanced tumors. A reasonable explanation may be that the antitumor effect of aspirin is realized by inhibiting the interaction between platelets and tumor cells, and thus fails to show obvious inhibitory effect on tumor cells that have undergone distant metastasis.

In general, although targeted interventions of platelet-tumor cell interactions have shown promising results in preclinical studies, evidence of clinical benefit remains limited except for aspirin. The randomized clinical trial of adjuvant aspirin after radical treatment is still in progress, which incorporates four phase III clinical randomized controlled studies. Patients with gastroesophageal cancer, colorectal cancer, breast cancer and prostate cancer were randomly given different doses of aspirin or placebo. The most common toxicity of grade 1–2 was indigestion, and no bleeding event was observed in the gastroesophageal cancer group, showing a good tolerance [129].

## Future perspectives

More and more evidences support the crucial role of platelets in tumor EMT and hematogenous metastasis. However, there are many questions need to be further clarified. It has been found that highly metastatic colorectal cancer cells, prostate cancer cells and breast cancer cells have stronger ability to stimulate platelet activation and aggregation compared with lowly metastatic cells [130]. Does greater platelet aggregation-inducing ability means stronger EMT plasticity? Do tumor cells in the EMT status from the primary site rely on platelet-derived factors to maintain EMT during circulation? Does continuous platelet activation lead to decreased platelet reactivity and declined ability to induce EMT? It has been found that reduced platelet count can suppress tumor growth in a mouse ovarian cancer model [131]. Hence, will platelet infusion increase the interaction between tumor cells and platelets and therefore raise the risk of hematogenous metastasis? At the same time, it is noted that platelets do not always contribute to tumor progression. PMPs has been observed to transport miR-24 to induce tumor cell apoptosis [93]. What’s more, platelet-treated fibrosarcoma cells show significant up-regulation of tumor suppressor genes [132]. Further understanding of the tumor-promoting and anti-tumor mechanisms of platelets will not only help us better understand the interaction between platelets and tumor cells, but also avail to the prevention and management of tumor patients. Although inhibitors have been proved to be a possible tumor prevention strategy, long-term medication, clinical benefits and side effects should be considered. Specific inhibition of pathological tumor cell-platelet interactions without interfering with normal platelet function is a reasonable direction.

## Data Availability

Not applicable.

## Abbreviations

EMT: Epithelial-mesenchymal transition
CAFs: Cancer-associated fibroblasts
TXA2: Thromboxane A2
TP: Thromboxane-prostanoid
PARs: Proteinase-activated receptors
PKC: Protein kinase C
PI3K: Phosphatidylinositol 3 kinase
PDPN: Podoplanin
CLEC-2: C-type lectin-like receptor 2
TGFβ: Transforming growth factor beta
TBK1: TANK-binding kinase 1
TCIPA: Tumor cell-induced platelet aggregation
PDGF: Platelet-derived growth factor
LPA: Lysophosphatidic acid
ATX: Autotaxin
RAGE: Receptor for advanced glycation end products
PMPs: Platelet-derived microparticles
MPs: Microparticles
S1P: Sphingosine 1-phosphate
GPVI: Glycoprotein VI
HRG: Histidine-rich glycoprotein
CMS: Consensus molecular subtypes
VTE: Venous thromboembolism
COX1: Cyclooxygenase-1
COX2: Cyclooxygenase-2
